# Oroxylin A ameliorates AKI-to-CKD transition through maintaining PPARα-BNIP3 signaling-mediated mitochondrial homeostasis

**DOI:** 10.3389/fphar.2022.935937

**Published:** 2022-08-23

**Authors:** Mengying Yao, Shaozong Qin, Jiachuan Xiong, Wang Xin, Xu Guan, Shuiqin Gong, Jing Chen, Yong Liu, Bo Zhang, Jinghong Zhao, Yinghui Huang

**Affiliations:** ^1^ School of Medicine, Chongqing University, Chongqing, China; ^2^ Department of Nephrology, The Key Laboratory for the Prevention and Treatment of Chronic Kidney Disease of Chongqing, Chongqing Clinical Research Center of Kidney and Urology Diseases, Xinqiao Hospital, Army Medical University (Third Military Medical University), Chongqing, China

**Keywords:** oroxylin A, acute kidney injury, mitochondrial energy metabolism, BNIP3, PPARα

## Abstract

**Background:** Acute kidney injury (AKI) occurs in approximately 7–18% of all hospitalizations, but there are currently no effective drug therapy for preventing AKI or delaying its progression to chronic kidney disease (CKD). Recent studies have shown that *Scutellaria baicalensis*, a traditional Chinese herb, could attenuate cisplatin-induced AKI, although the mechanism remains elusive. Further, it is unknown whether its major active component, Oroxylin A (OA), can alleviate kidney injury.

**Methods:** The therapeutic effect of OA was evaluated by using ischemia-reperfusion (IR) and cisplatin mediated-AKI mice and HK-2 cells under hypoxia-reoxygenation (HR) conditions. HE staining, transmission electron microscopy, flow cytometry, immunofluorescence, qPCR, Western blot, PPARα inhibitor, BNIP3 siRNA and ChIP assay were used to explore the role and mechanism of OA in AKI.

**Results:** OA ameliorated tubular damage and dramatically decreased serum creatinine (Scr) and urea nitrogen (BUN), and the expressions of renal injury markers (Kim-1, Ngal) in AKI mice induced by both IR injury and cisplatin, as well as attenuating AKI-to-CKD transition. *In vitro* experiments showed that OA alleviated HR-induced mitochondrial homeostasis imbalance in renal tubular epithelial cells. Mechanistically, OA dose-dependently induced the expression of Bcl-2/adenovirus E1B 19-kDa interacting protein (BNIP3), while knockdown of BNIP3 expression reversed the protection of OA against HR-mediated mitochondrial injury. Network pharmacological analysis and experimental validation suggested that OA enhanced BNIP3 expression via upregulating the expression of peroxisome proliferator activated receptor alpha (PPARα), which induced the transcription of BNIP3 via directly binding to its promoter region. Both *in vitro* and *in vivo* experiments confirmed that the renoprotective effect of OA was dramatically reduced by GW6471, a PPARα antagonist.

**Conclusion:** Our findings revealed that OA ameliorates AKI-to-CKD transition by maintaining mitochondrial homeostasis through inducing PPARα-BNIP3 signaling pathway, indicating that OA may serve as a candidate therapeutic strategy for alleviating AKI and CKD.

## Introduction

Acute kidney injury (AKI) is a common critical illness, characterized by a sharp decrease of glomerular filtration rate, accompanied by retention of nitrogen products (such as creatinine and urea nitrogen) (Bellomo et al., 2012; Levey and James, 2017; Ronco et al., 2019). AKI is also one of the significant risk factors for progressive chronic kidney disease (CKD) and end-stage renal disease (ESRD) (Chawla et al., 2014; Kramann et al., 2014; Chen et al., 2019). Although the kidney can gradually recover from AKI through regenerative capacity and repair, accumulating evidence suggests that there is a high risk of developing CKD after AKI (Bucaloiu et al., 2012; Coca et al., 2012; Wu et al., 2014). Yet, no effective drug is clinically applicable for AKI or AKI-to-CKD transition. Therefore, novel therapeutic strategies to alleviate AKI are highly desired.

Traditional Chinese medicine reveals that chronic renal failure is located in the spleen and kidney, and the pathogenesis can be summarized as deficiency of the yang qi of spleen, while kidney is the cause and the internal stagnation of dampness, turbidity, stasis and toxin is the symptom (Liu et al., 2014). Recently, traditional Chinese medicine suggests that herbal medicines that are capable of invigorating the spleen and kidney, promoting blood circulation, and removing turbidity may be effective in the treatment of CKD and renal fibrosis (Zhao et al., 2022). *Scutellaria baicalensis* Georgi (SB), a traditional Chinese medicine, has been prescribed to treat diseases in China for thousands of years, such as inflammation (Guan et al., 2020) and fibrosis (Kong et al., 2011; Pan et al., 2012), even though the mechanism remains elusive. SB can alleviate chemotherapy-induced AKI and protect against CKD (Huang et al., 2019; Guan et al., 2020). Simultaneously, *baicalenin* (a component of SB) can protect the kidney injury induced by renal ischemia-reperfusion injury (IRI) (Lin et al., 2014) or myocardial IR (Lai et al., 2016). Oroxylin A (OA), the main active component of SB, can alleviate lipopolysaccharide (LPS)-mediated acute injury of liver and lung (Tseng et al., 2012; Huang et al., 2015). However, the role and mechanism of OA in the process of AKI-to-CKD transition remain unclear.

Given the function of the kidney to remove waste from the blood by filtration and regulate fluid and electrolyte balance, it is destined to consume a large amount of energy, which is mainly provided by mitochondrial fatty acid β-oxidation (FAO) (Bhargava and Schnellmann, 2017; Sun et al., 2019). Mitochondrial energy metabolism is one of the important components of mitochondrial homeostasis, and maintaining mitochondrial homeostasis will benefit for the restoration of renal function (Emma et al., 2016; Bhargava and Schnellmann, 2017; Sun et al., 2019; Tang et al., 2021). Mitochondrial damage in proximal tubule cells has gradually evolved as a feature of various forms of AKI (Parikh et al., 2015; Sun et al., 2019). Accumulating evidences, including ours, suggested mitochondrial injury as a major contributor to acute and chronic kidney disease (Huang et al., 2020a; Huang et al., 2020b; Yu et al., 2020; Huang et al., 2022). Therefore, exploring therapeutic strategies aimed at restoring mitochondrial homeostasis may hold great potential for improving renal function (Emma et al., 2016; Bhargava and Schnellmann, 2017; Sun et al., 2019; Tang et al., 2021).

In the present study, based on the effect of OA in alleviating acute liver injury and acute lung injury (Tseng et al., 2012; Huang et al., 2015), the therapeutic potential of OA in AKI was evaluated and its molecular mechanism was explored. Our findings suggest that OA maintains mitochondrial homeostasis *via* inducing peroxisome proliferator activated receptor alpha (PPARα)-Bcl-2/adenovirus E1B 19-kDa interacting protein (BNIP3) signaling pathway, thereby alleviating AKI and its progression to CKD.

## Materials and methods

Additional details for all methods are provided in the Supplementary Methods.

### Materials

Oroxylin A (OA) and Cisplatin were obtained from MedChemExpress (MCE, Monmouth Junction, NJ, United States). GW6471 (PPARα antagonist) and WY-14643 (PPARα agonist) were bought from Selleckchem (Houston, TX, United States).

### Animal study

Male C57BL/6J mice (8-week-old) were purchased from Chongqing Tengxin Bioscience (Chongqing, China). The construction of IRI-induced AKI mouse model was previously described (Hu et al., 2017).

### Cell treatment

For the hypoxia-reoxygenation (HR) model, HK-2 cells were incubated in a hypoxic incubator at 1% O_2_ for 24 h, and then the cells were put back into the normal incubator and reoxygenated for 6 h. After treatment with OA, the cells were harvested for the subsequent analysis.

### Transmission electron microscopy observation

The collected HK-2 cells and mouse kidney tissue were immersed in a transmission electron microscopy solution containing 2.5% glutaraldehyde and 4% paraformaldehyde, and the samples were treated in the transmission electron microscopy room of the Central Laboratory of Army Medical University. The microstructure of mitochondria was observed using TEM (JEM-1400PLUS, Japan).

### Statistical analysis

All data were presented as mean ± SEM. Unpaired *t* test or one-way analysis of variance (ANOVA) with Tukey’s test were used for statistical analysis. Statistical significance was defined as *P* < 0.05.

## Results

### Oroxylin A alleviates ischemic-reperfusion and cisplatin-induced acute kidney injury *in vivo*

**FIGURE 1:**
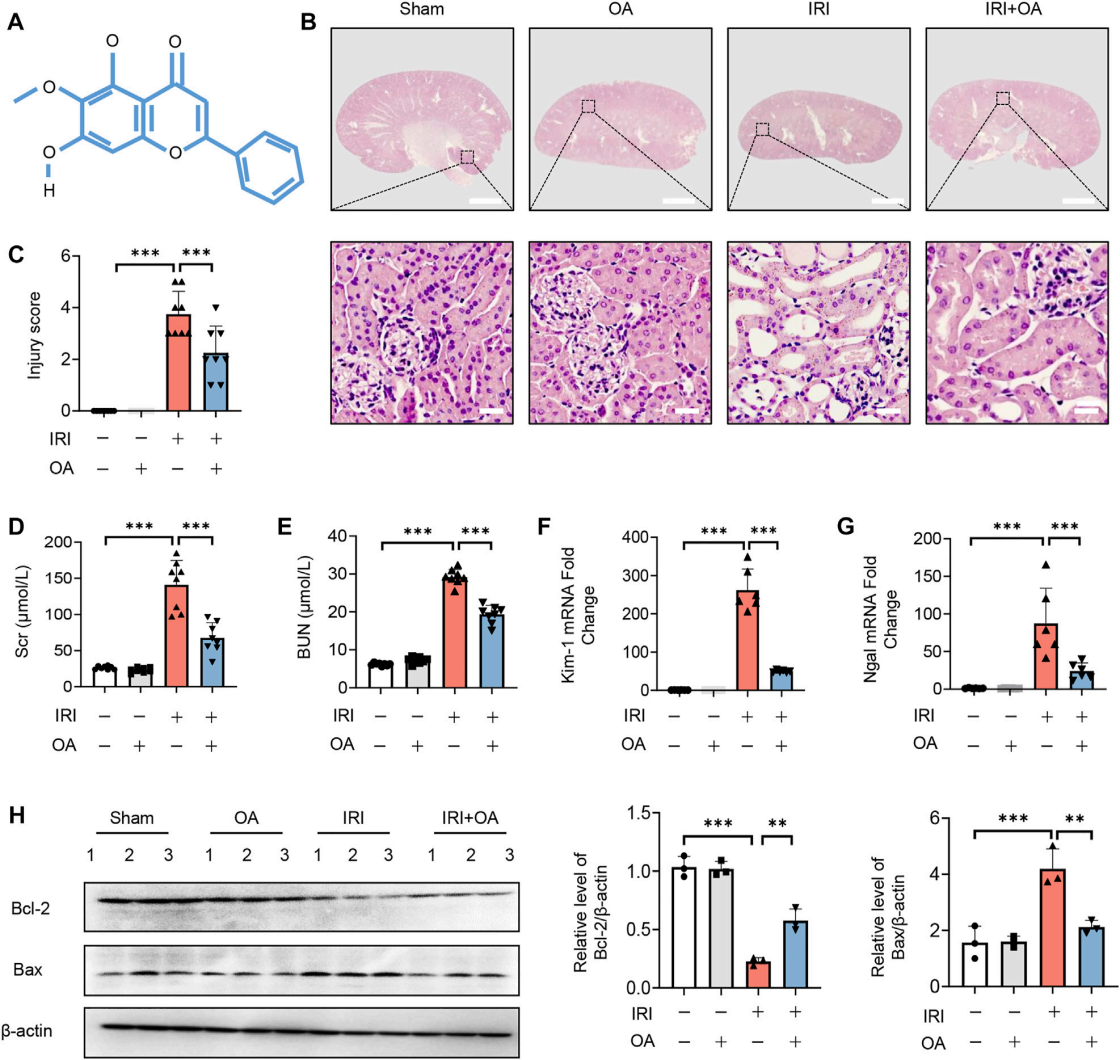
OA alleviates IRI-induced AKI *in vivo*. **(A)** The chemical structure of OA. **(B)** Representative micrographs of HE staining of kidney sections from sham and IRI-induced AKI mice intravenously injected with control or OA. Scale bars, 1.25 mm (top) and 50 μm (bottom). **(C)** Scoring of injury score according to HE staining of kidney sections from mice in **(A)** (*n* = 8). Effects of OA on Scr **(D)** and BUN **(E)** of mice in **(A)** (*n* = 8). The mRNA levels of Kim-1 **(F)** and Ngal **(G)** were analyzed by qPCR (*n* = 8). **(H)** The expressions of Bcl-2 and Bax in kidney tissues from mice in **(A)** were analyzed by Western blot (*n* = 3). Data are expressed as means ± SEM. ∗∗*P* < 0.01, ∗∗∗*P* < 0.001.

The chemical formula of OA was shown in Figure 1A. To determine the therapeutic effect of OA, OA (20 mg/kg) was intravenously injected into sham and AKI mice. HE staining showed that OA improved renal damage in AKI mice (Figures 1B,C). In AKI mice, the levels of Scr, BUN and the expressions of kidney injury markers (Kidney injury molecule-1, Kim-1; and Neutrophil gelatinase-associated lipocalin, Ngal) and apoptosis-related proteins, including B-cell lymphoma-2 (Bcl-2) and Bcl-2-associated x (Bax), were markedly elevated, which were significantly ameliorated by OA treatment (Figures 1D–H). Meanwhile, we also found that OA alleviated cisplatin-induced renal tubular damage, increased levels of Scr and BUN, and upregulated expressions of Kim-1 and Ngal (Supplementary Figures 1A–E). These data suggest that OA can ameliorate IR and Cisplatin-induced AKI.

### Oroxylin A attenuates hypoxia-reoxygenation-Mediated mitochondrial homeostasis imbalance *in vitro*

**FIGURE 2:**
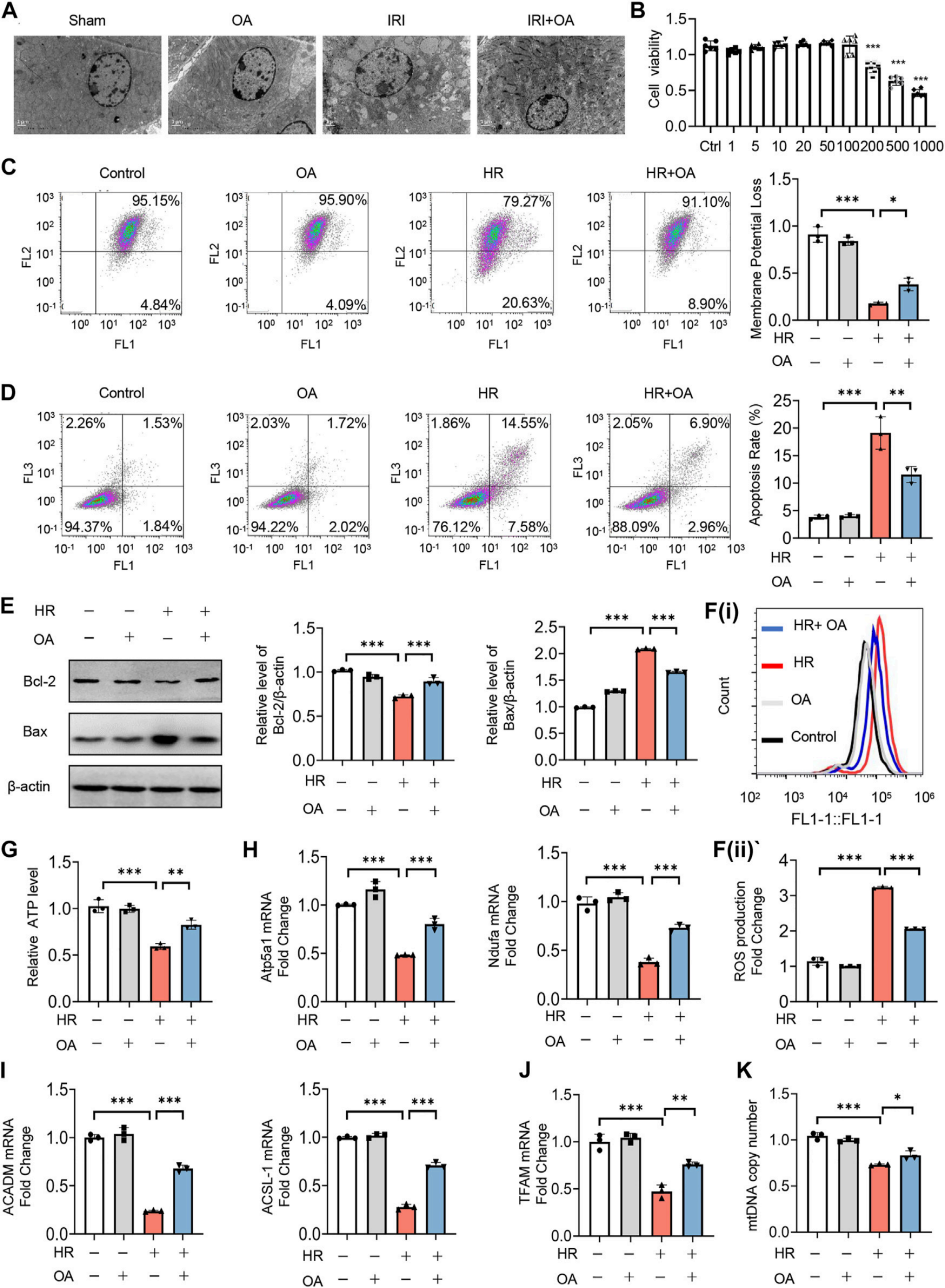
OA ameliorates hypoxia-reoxygenation (HR)-mediated mitochondrial homeostasis imbalance *in vitro*. **(A)** Representative TEM images of kidney tissues from sham and AKI mice treated with control or OA (20 mg/kg) for 24 h. Scale bar, 1 μm. **(B)** Cell viability of HK-2 cells incubated with indicated concentrations of OA for 48 h was determined using CCK-8. **(C**–**I)** HK-2 cells under HR exposure were treated with control or OA (10 μM), then the mitochondrial membrane potential **(C)**, apoptosis **(D**,**E)**, ROS **(F)**, ATP **(G)**, the expressions of Atp5a1, Ndufa, ACADM and ACSL-1 **(H**,**I)** were analyzed (*n* = 3). **(J**,**K)** HK-2 cells under HR exposure were treated with control or OA (10 μM), the expression of TFAM **(J)** and relative mitochondrial DNA copy number **(K)** were analyzed by qPCR (*n* = 3). Data are expressed as means ± SEM. ns: no significance. ∗*P <* 0.05, ∗∗*P* < 0.01, ∗∗∗*P* < 0.001.

Subsequently, we analyzed the kidney tissues by TEM observation, and found that mitochondrial morphological damage, including mitochondrial swelling and vacuolation with disorganized or fragmented cristae, and the accumulation of lipid droplets in AKI mice were significantly improved after OA treatment (Figure 2A). Meanwhile, the decreased expressions of mitochondrial oxidative phosphorylation (OXPHO)-related genes (synthase H^+^ transporting mitochondrial F1 complex alpha subunit 1, Atp5a1; NADH ubiquinone oxidoreductase alpha subunit, Ndufa; NADH ubiquinone oxidoreductase flavoprotein, Ndufv and Cytochrome c, Cytc) and fatty acid oxidation (FAO)-related genes (Carnitine palmityl transferase 1b, Cpt1b; Long Chain Acyl Coenzyme A Synthetase 1, ACSL-1; O-octanoyltransferase, CROT; Acyl Coenzyme A Dehydrogenase Medium chain, ACADM) and lipid deposition in AKI mice were also restored by OA (Supplementary Figures 2A–C). Further, CCK-8 assays demonstrated that OA did not inhibit the viability of HK-2 cells, when its concentration was less than 100 μM (Figure 2B). To verify the effect of OA *in vitro*, HK-2 cells were incubated with OA (10 μM) after HR treatment. It was found that OA could alleviate mitochondrial damage, as evidenced by reducing apoptosis and ROS production, as well as restoring mitochondrial membrane potential, ATP level and the expression levels of OXPHO- and FAO-related gene (Figures 2C–I). Further, OA attenuated HR-mediated disorder of mitochondrial biogenesis, as evidenced by downregulated transcription factor A, mitochondrial (TFAM) expression and reduced mtDNA copy number (Figures 2J,K). These findings reveal that OA improves HR-induced mitochondrial morphological and functional damage.

### Oroxylin A ameliorates mitochondrial homeostasis *via* upregulating BNIP3 expression

**FIGURE 3:**
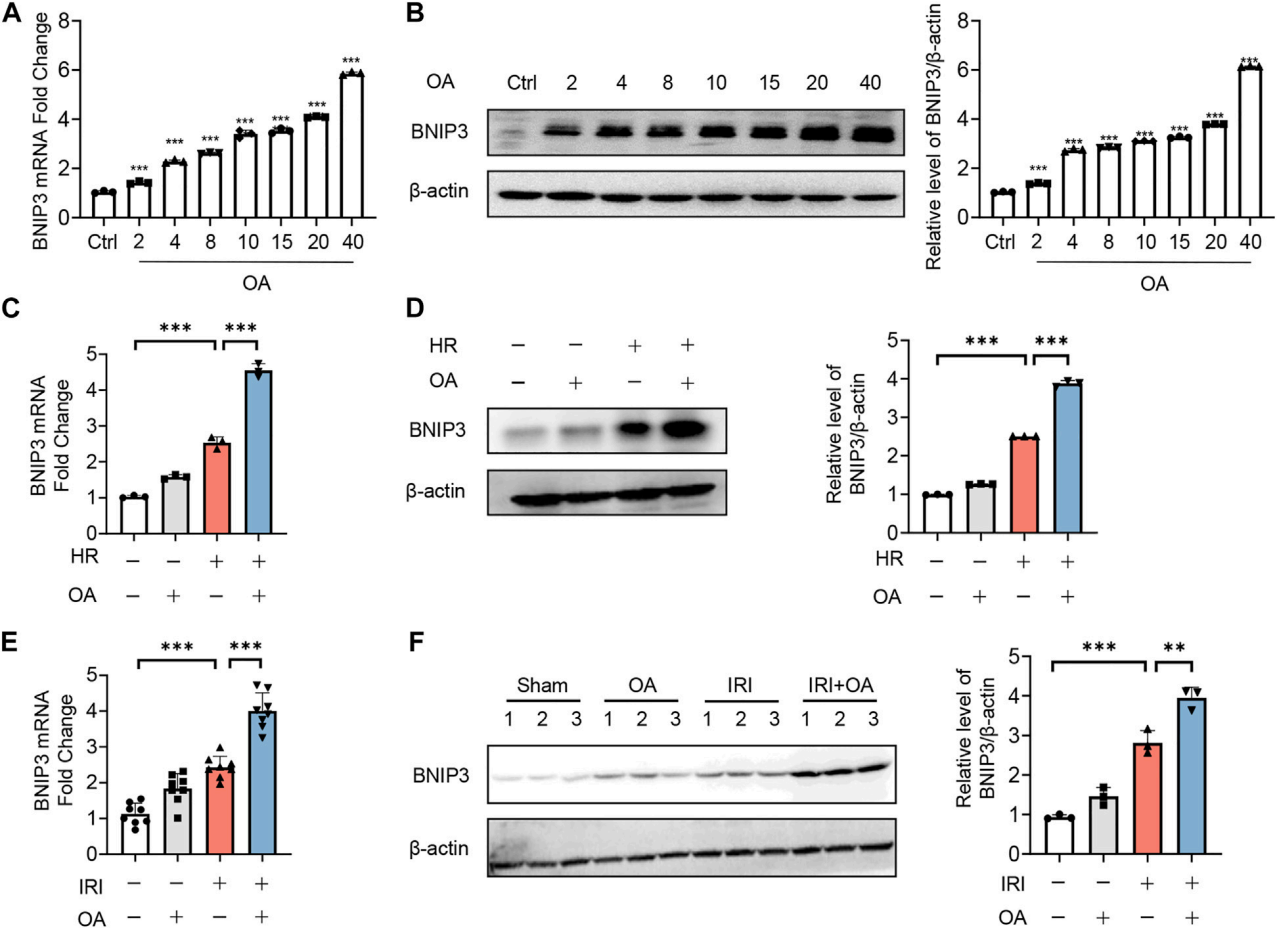
OA enhances BNIP3 expression *in vitro* and *in vivo*. **(A**,**B)** qPCR **(A)** and Western blot **(B)** analysis of the expression of BNIP3 in HK-2 cells treated with OA for 24 h (*n* = 3). **(C**,**D)** The mRNA and protein expression of BNIP3 in HK-2 cells exposed to HR followed by incubation with control or OA were analyzed by qPCR **(C)** and Western blot **(D)** (*n* = 3). **(E**,**F)** qPCR **(E**, *n* = 8**)** and Western blot **(F**, *n* = 3**)** analysis of the expression of BNIP3 in kidney tissues from sham and AKI mice treated with control or OA. Data are expressed as means ± SEM. ∗∗*P* < 0.01, ∗∗∗*P* < 0.001.

Since recent studies reported that BNIP3 was essential for mitochondrial quality control and bioenergetics (Choi et al., 2016), we evaluated its role in OA-mediated effects. We found that OA dose-dependently induced BNIP3 expression at both transcriptional and translational level (Figures 3A,B). Simultaneously, BNIP3 expression was further enhanced in both OA-treated cells exposed to HR and kidney tissues from AKI mice (Figures 3C–F).

**FIGURE 4:**
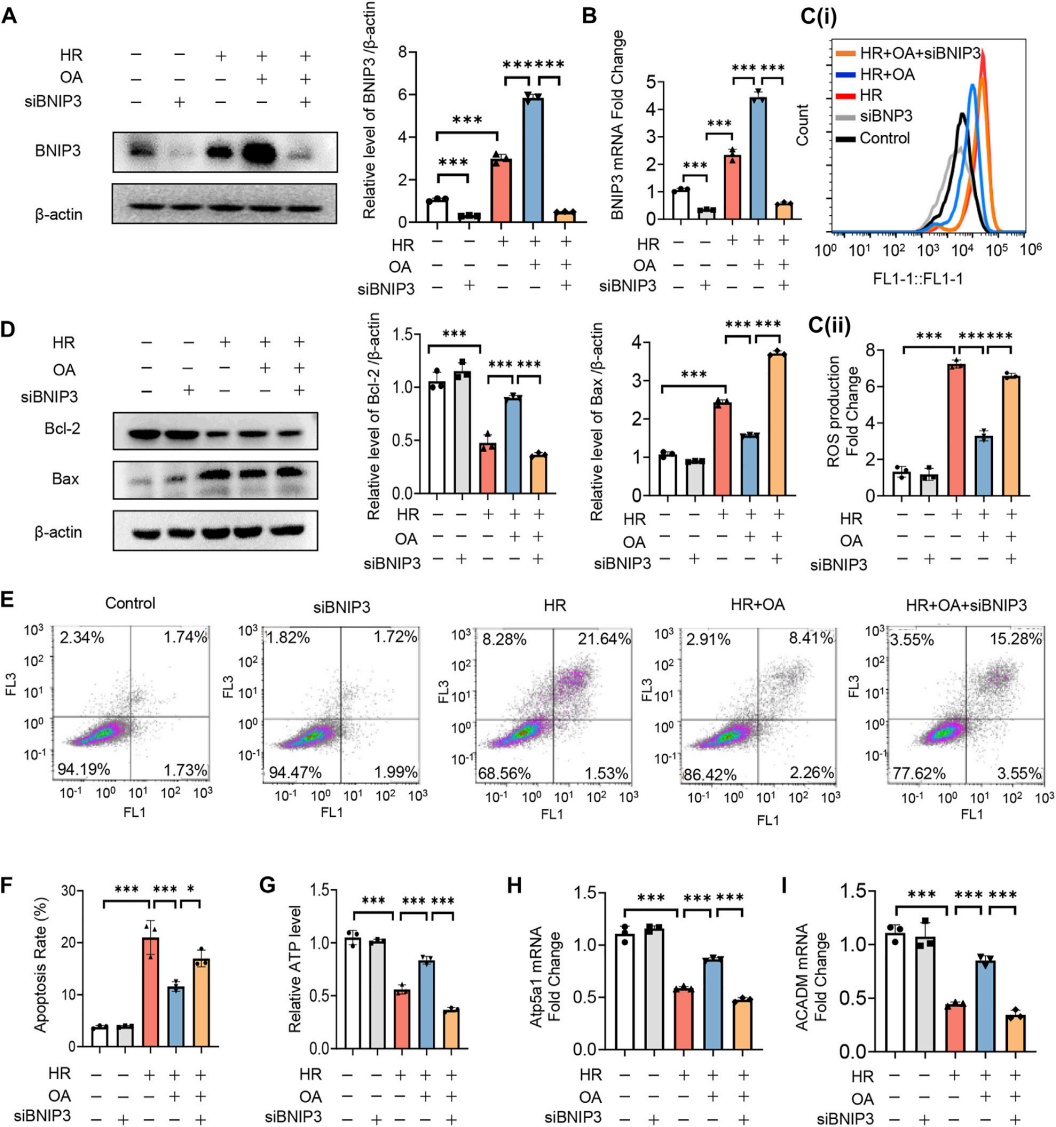
OA improves mitochondrial homeostasis imbalance through inducing BNIP3 expression. **(A**–**I)** After 24 h transfection with siBNIP3, HK-2 cells were exposed to HR and treated with control or OA. The protein expression level of BNIP3 **(A)**, the levels of BNIP3 mRNA **(B)**, ROS **(C)**, apoptosis **(D**–**F)**, ATP **(G)**, the expression levels of OXPHO-related gene **(H)** and FAO-related gene **(I)** were detected (*n* = 3). Data are expressed as means ± SEM. ∗*P <* 0.05, ∗∗∗*P* < 0.001.

To verify whether OA maintains mitochondrial homeostasis by inducing BNIP3 expression, we first transfected siRNAs against BNIP3 (siBNIP3) into HK-2 cells, and then treated the cells with HR model (Figures 4A,B). We found that OA cannot improve mitochondrial injury when BNIP3 was silenced, as evidenced by increased ROS production and apoptosis, decreased ATP levels, and downregulated expressions of OXPHO-related gene Atp5a1 and FAO-related gene ACADM (Figures 4C–I). As BNIP3 is well known for mitophagy induction that can protect against AKI (Fu et al., 2020), we also investigated the role of mitophagy after OA treatment. We treated HK-2 cells exposed to normoxia or HR injury with control or OA. However, the Western blot assay and immunofluorescence observations showed that OA neither induced mitophagy under normoxia conditions nor aggravated HR-mediated mitophagy (Supplementary Figures 3A–C). Further, OA could not regulate mitophagy in AKI mice either (Supplementary Figure 3D). These results collectively indicate that OA maintains mitochondrial homeostasis through inducing the expression of BNIP3 without regulating mitophagy in renal tubular epithelial cells.

### Transcription factor PPARα induces BNIP3 expression *via* directly binding to its promoter region

**FIGURE 5:**
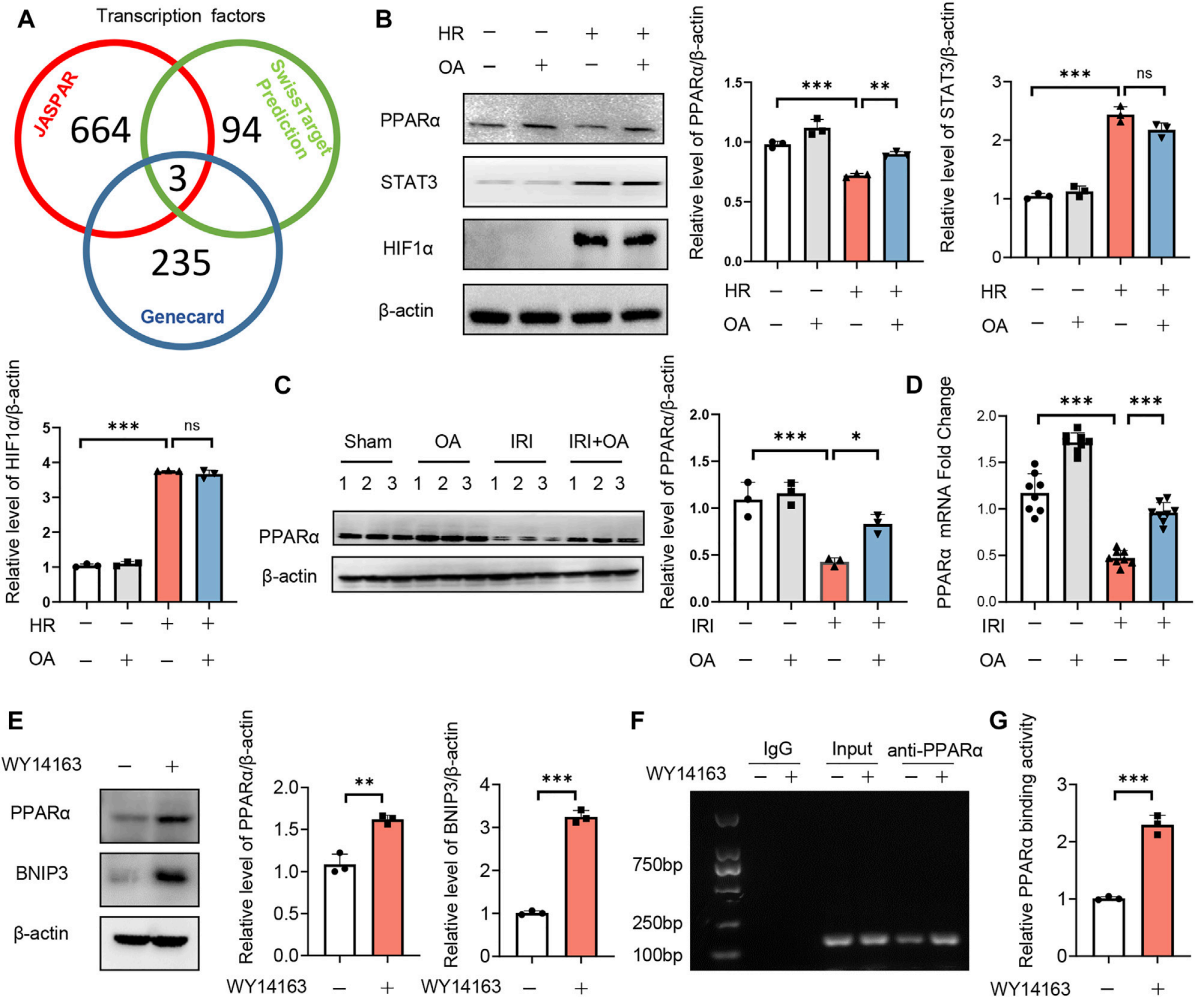
Transcription factor PPARα induces BNIP3 expression via directly binding to its promoter region. **(A)** JASPAR, Genecard and SwissTarget (network pharmacology analysis) were employed to predict the upstream transcription factors of BNIP3, and three potential transcription factors were obtained, including PPARα, STAT3 and HIF-1α. **(B)** Expression of PPARα, STAT3, and HIF-1α in HK-2 cells incubated with control or OA after HR treatment (*n* = 3). **(C**,**D)** Expression of PPARα in kidney tissue from sham and AKI mice treated with control or OA (*n* = 3–8). **(E)** Expressions of PPARα and BNIP3 in HK-2 cells treated with Control or PPARα agonist (*n* = 3). **(F**,**G)** The binding ability of PPARα to BNIP3 promoter region was assayed by ChIP (*n* = 3). Data are expressed as means ± SEM. ns: no significance. ∗∗*P* < 0.01, ∗∗∗*P* < 0.001.

To investigate the upstream transcription factors of BNIP3, JASPAR, Genecard and SwissTarget prediction (network pharmacology analysis) were used, which predicted three potential transcription factors, including PPARα, signal transducer and transcription 3 (STAT3) and hypoxia-inducible factor 1 activator of subunit alpha (HIF-1α) (Figure 5A). After screening, we found that only the expression of PPARα was restored by OA in HK-2 cells exposed to HR and kidney tissues from AKI mice (Figures 5B–D). The expressions of PPARα and BNIP3 were also enhanced by a PPARα agonist, WY14163 (Figure 5E). Further, ChIP assay demonstrated that PPARα could induce BNIP3 transcription *via* directly binding to its promoter region (Figures 5F,G).

### Oroxylin A restores mitochondrial homeostasis by enhancing PPARα-BNIP3 signaling pathway

**FIGURE 6:**
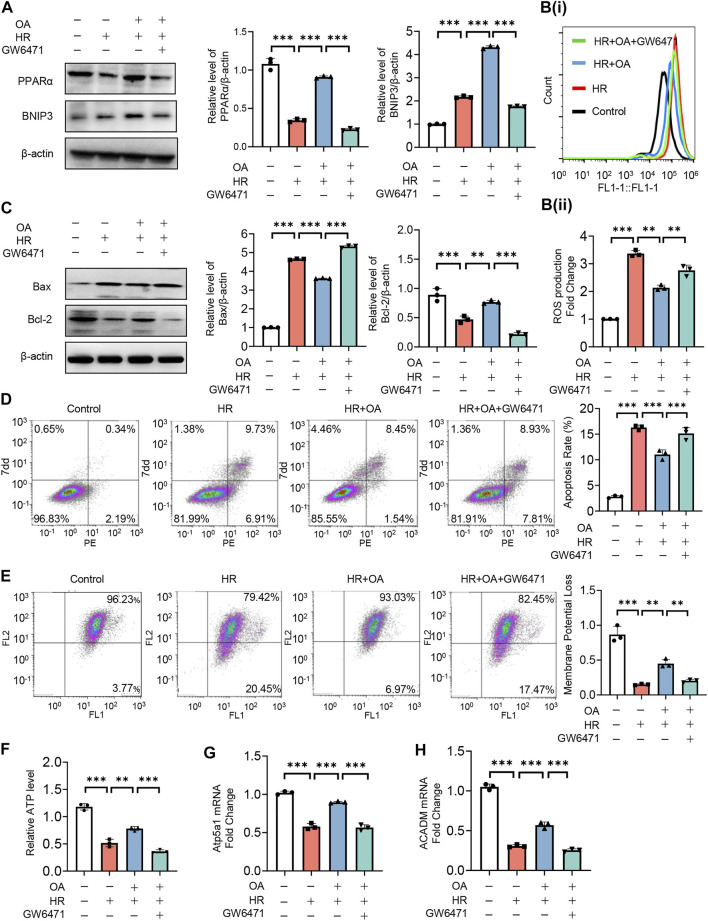
OA alleviates mitochondrial homeostasis imbalance by enhancing PPARα-BNIP3 signaling pathway. **(A**–**H)** HK-2 cells were treated with OA and control or GW6471 (PPARα antagonist) under normal or HR injury conditions, and then the levels of PPARα and BNIP3 expressions **(A)**, ROS **(B)**, apoptosis **(C**,**D)**, mitochondrial membrane potential **(E)**, ATP **(F)**, OXPHO-related **(G)** and FAO-related gene expressions **(H)** were determined (*n* = 3). Data are expressed as means ± SEM. ∗∗*P* < 0.01, ∗∗∗*P* < 0.001.

To further verify the role of PPARα, we treated HK-2 cells under normal or HR injury with OA in combined with or without GW6471, a PPARα antagonist. We found that OA cannot upregulate the expressions of PPARα and BNIP3 after the co-incubation with GW6471 (Figure 6A). Meanwhile, OA did not alleviate mitochondrial damage when PPARα expression was inhibited, as evidenced by elevated ROS production and apoptosis, repressed mitochondrial membrane potential and ATP levels, and suppressed expressions of Atp5a1 and ACADM (Figures 6B–H). These results collectively suggest that OA restores mitochondrial homeostasis through enhancing PPARα-BNIP3 signaling pathway.

### PPARα inhibition reversed the protective effect of Oroxylin A in acute kidney injury mice

**FIGURE 7:**
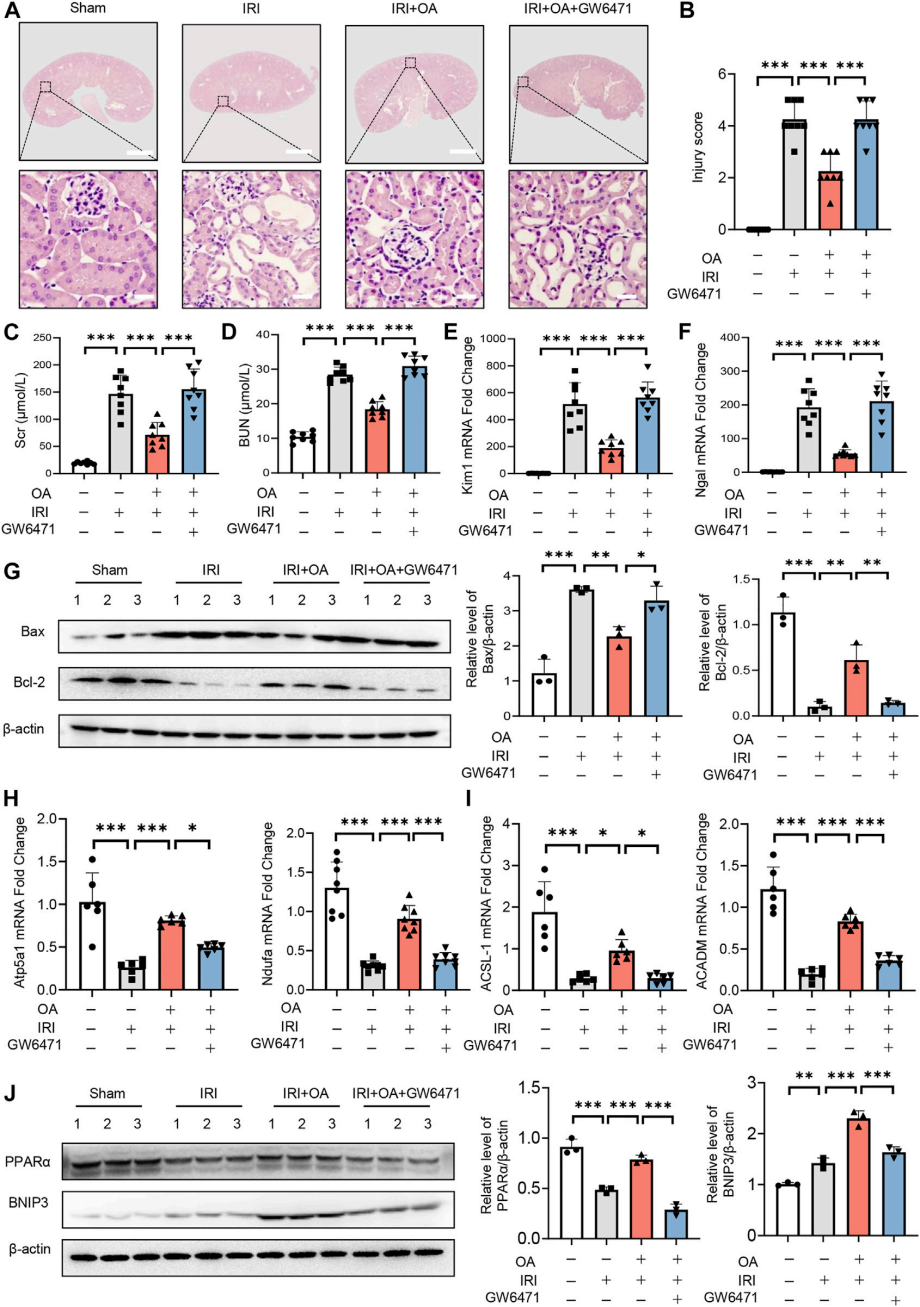
PPARα inhibition reversed the protective effect of OA in AKI mice. **(A)** OA-treated Sham and AKI mice were injected with control or PPARα antagonist. Representative HE staining images of kidney sections from sham and AKI mice treated with control or OA in combination with or without PPARα antagonist. Scale bars, 1.25 mm (top) and 50 μm (bottom). **(B)** Quantitative assessment of tubular damage (*n* = 8). Effects of OA on serum Scr **(C)** and BUN **(D)** (*n* = 8). mRNA levels of Kim-1 **(E)** and Ngal **(F)** by qPCR (*n* = 8). **(G)** The expressions of Bcl-2 and Bax in kidney tissues were analyzed by Western blot analysis (*n* = 3). **(H**,**I)** The expression levels of OXPHO-related gene **(H)** and FAO-related gene **(I)** (*n* = 8) were detected by qPCR. **(J)** The expression levels of PPARα and BNIP3 in kidney tissues were measured by Western blot (*n* = 3). Data are expressed as means ± SEM. ∗*P <* 0.05, ∗∗*P* < 0.01, ∗∗∗*P* < 0.001.

To analyze the *in vivo* effect of PPARα, OA-treated AKI mice were injected with control or GW6471. HE staining exhibited that OA did not ameliorate renal tubular injury in AKI mice, when PPARα expression was inhibited (Figures 7A,B). Consistent with the histological findings, OA could not restore the serum levels of in Scr, BUN, the expressions of kidney injury markers (Kim-1 and Ngal), apoptosis-related genes (Bax and Bcl-2), OXPHO-related genes (Atp5a1 and Ndufa) and FAO-related genes (ACSL-1 and ACADM), or BNIP3 in AKI mice (Figures 7C–J).

### Oroxylin A improves the transition of acute kidney injury-to-chronic kidney disease *in vivo*

**FIGURE 8:**
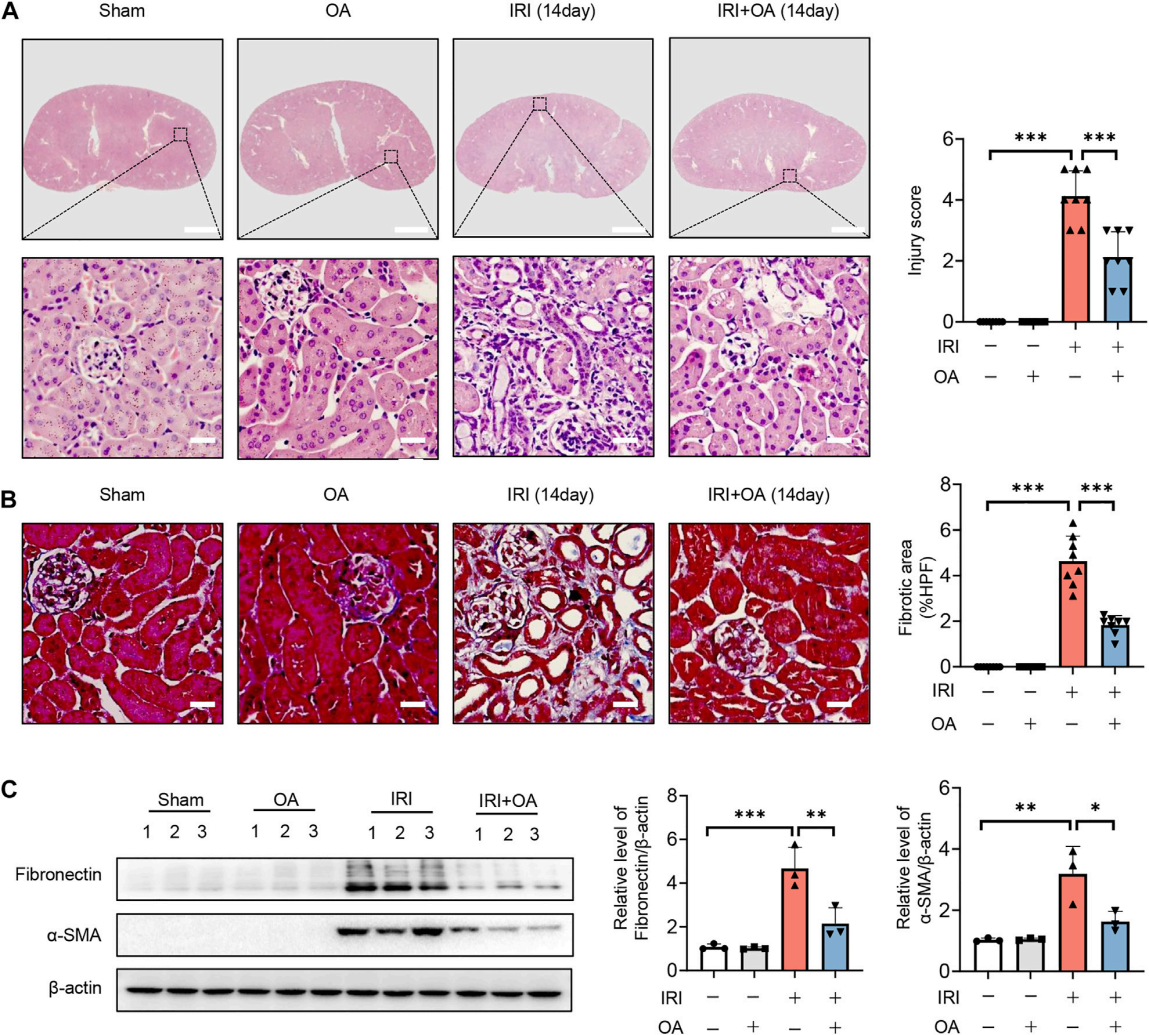
OA attenuates the transition of AKI-to-CKD. A 35-min bilateral IRI-induced AKI-to-CKD mouse model was constructed, which was intravenously injected with control or OA (20 mg/kg). Mice were euthanized 2 weeks later. **(A)** Representative images of HE staining. Scale bars, 1.25 mm (top) and 50 μm (bottom). **(B)** Representative images of Masson staining. Scale bar, 50 μm. **(C)** Western blot analysis of the expressions of fibrosis markers (Fibronectin and α-SMA) in the kidney tissues (*n* = 3). Data are expressed as means ± SEM. ∗*P <* 0.05, ∗∗*P* < 0.01, ∗∗∗*P* < 0.001.

To further confirm the role of OA, AKI-to-CKD transition mouse model was constructed. HE and Masson staining showed that tubulointerstitial injury and fibrosis were significantly improved after OA treatment (Figures 8A,B). Besides, we also found that the expressions of fibrosis markers, including fibronectin and α-smooth muscle actin (α-SMA), were markedly decreased by OA in the kidney tissues from IRI mice (Figure 8C). These findings reveal that OA could ameliorate the progression of AKI to CKD.

## Discussion

AKI remains a difficult clinically critical disease, with high morbidity and mortality (Bellomo et al., 2012; Levey and James, 2017; Ronco et al., 2019). Although significant progression has been made in the pathogenesis, there is still no drugs applicable for curing AKI in clinical practice (Hong et al., 2021; Kim et al., 2021), which highlights the urgent necessity to develop novel therapeutic strategies for AKI.

Although emerging studies support the beneficial role of OA in the pathogenesis of LPS-mediated acute injury of liver and lung (Tseng et al., 2012; Huang et al., 2015), the therapeutic role of OA in ischemic AKI is unclear. This study has identified the ability of OA in ameliorating AKI and delaying its transition to CKD. It was found that OA alleviated IRI and Cisplatin-induced renal tubular damage. In addition, at 14 days post-35 min bilateral renal IRI, mouse kidneys developed fibrosis and progressively progressed to CKD, which was prevented by the administration of OA. Further, OA significantly ameliorated HR-mediated mitochondrial injury via inducing PPARα-BNIP3 signaling pathway, which may shed new light on the treatment of AKI and its progression to CKD.

The kidney is a human organ with the second highest mitochondrial abundance, which plays a vital role in maintaining kidney health (Wang et al., 2020). Increasing evidence demonstrated mitochondrial injury as a crucial cause of kidney diseases (Huang et al., 2020a; Huang et al., 2020b; Scholz et al., 2021), while maintaining mitochondrial homeostasis may improve kidney injury (Szeto et al., 2016; Bhargava and Schnellmann, 2017). In this study, we found that OA alleviated mitochondrial damage through inducing the expression of BNIP3. BNIP3 is localized in the mitochondrial outer membrane, playing a vital role in mitochondrial quality control (Chourasia et al., 2015; Lee et al., 2017; Tang et al., 2019; Fu et al., 2020). Recent studies have reported that the loss of BNIP3 aggravated IR-induced AKI, while overexpression of BNIP3 not only reversed the reduction of mitophagy, but also alleviated renal injury, indicating that BNIP3-mediated mitophagy plays a protective role in AKI (Tang et al., 2019; Fu et al., 2020). However, in the present study, we found that OA attenuated AKI without regulating mitophagy in the kidney, which coincided with other reports that mitophagy can also be induced independent of BNIP3 (Choi et al., 2016, Yuan and Pan, 2018). In addition, BNIP3 ensured mitochondrial quality and integrity *via* regulating FAO in the liver (Glick et al., 2012; Choi et al., 2016), reflecting a new role of BNIP3 in mitochondrial quality control. We demonstrated that OA attenuated HR-mediated mitochondrial injury, including mitochondrial energy metabolism remodeling, through upregulating BNIP3 expression in renal tubular epithelial cells. In-depth *in vivo* studies using kidney-specific BNIP3 knockout mice may help validate the *in vitro* findings and overcome this limitation. Despite of this, our results together with previous reports collectively suggest that therapeutic approaches targeting BNIP3-mediated mitochondrial homeostasis may develop as a promising strategy for the treatment of AKI.

Further network pharmacological analysis, transcription factor prediction and experimental validations suggested that OA enhanced BNIP3 expression via upregulating the expression of PPARα, which induced BNIP3 transcription via directly binding to its promoter region. PPARα is a subtype of ligand-activated transcription factor, which has been shown mainly distributed in tissues with a high FAO, such as kidney and brown fat (Pawlak et al., 2015). PPARα plays a crucial role in lipid metabolism by regulating various target genes involved in FAO and lipoprotein metabolism (Nan et al., 2014; Pawlak et al., 2015; Yu et al., 2015). 90% of the ATP required by proximal tubule cells in the renal cortex is produced by mitochondrial FAO (Console et al., 2020), which is an essential part of mitochondrial energy metabolism. As reported, FAO decreases during hypoxia, exhibiting the same phenotype as renal fibrosis, which gradually leads to CKD and ESRD (Console et al., 2020). In this study, we observed that PPARα expression was significantly upregulated in OA-treated cells under HR conditions and in kidney tissues from AKI mice. Of note, when PPARα expression was inhibited, OA could neither ameliorate HR-induced mitochondrial homeostasis imbalance *in vitro*, nor IRI-mediated AKI *in vivo*, further supporting the regulatory effect of OA on PPARα. Therefore, OA-induced PPARα-BNIP3 axis-mediated mitochondrial homeostasis may serve as a novel target for AKI treatment.

As reported, OA possesses multi-pharmacological activities not only restricted in anti-tumor effects, but also in anti-inflammation and neuro-protection (Lu et al., 2016). In carcinoma, OA could increase ROS level, induce apoptosis, suppress angiogenesis and enhance mitochondrial dysfunction via inactivating sirtuin 1 (SIRT1)-forkhead box O3 (FOXO3)-BNIP3 axis (Lu et al., 2016; Yao et al., 2021). However, in normal tissues, OA plays a protective effect through inhibiting mitochondrial ROS, repressing inflammatory response, and promoting angiogenesis (Lu et al., 2016; Kai et al., 2020; Zhang et al., 2021). Recently, a literature review compared the inconsistent roles of OA in tumors and normal tissues, and found the high selectivity of OA due to the unfolded protein response and protein kinase B pathway, since OA (100–200 μM) selectively killed many more hepatocellular carcinoma cells (HepG2) than normal hepatocytes (L02) (Mu et al., 2009; Xu et al., 2012; Lu et al., 2016). Further, the concentrations of OA used in tumor treatment were mainly 40–200 μM (Lu et al., 2016), while the therapeutic dose of OA was 10 μM in the present study. As shown in Figure 2B, OA could not inhibit the viability of HK-2 cells, when its concentration was less than 100 μM. Therefore, the different roles and underlying mechanisms of OA may be attributed to tissue specificity and various dosages, and low doses of OA may improve tumor progression while protecting normal cells.

## Conclusion

The present study demonstrated the therapeutic effect of OA, the main active component of a traditional Chinese medicine SB, on AKI and its progression to CKD. Mechanistically, OA significantly improved HR-induced mitochondrial injury via enhancing PPARα-BNIP3 signaling pathway. Therefore, therapeutic approaches using OA or targeting PPARα-BNIP3 axis to modulate mitochondrial homeostasis may provide novel strategies for the treatment of AKI-to-CKD transition.

## Data Availability

The original contributions presented in the study are included in the article/Supplementary Materials, further inquiries can be directed to the corresponding authors.
