# Supplementary material for: Oroxylin A ameliorates AKI-to-CKD transition through maintaining PPARα-BNIP3 signaling-mediated mitochondrial homeostasis
Source: Front Pharmacol. 2022 Aug 23;13:935937. doi: 10.3389/fphar.2022.935937 (PMC9445212; doi:10.3389/fphar.2022.935937)
Supplement: Supplementary file 1 [file DataSheet1.pdf]

# Supplementary Materials

**This PDF file includes:**

**1. Supplementary Materials and Methods**

**2. Supplementary Figures**

**3. Supplementary Tables**

## 1. Supplementary Materials and Methods

### IRI and Cisplatin induced mouse models

Male C57BL/6J mice (8-week-old) were purchased from Chongqing Tengxin Bioscience (Chongqing, China). The construction of IRI-induced AKI mouse model was previously described (Hu et al., 2017). Brifely, mice were placed under general anesthesia for laparotomy, and their bilateral renal infarcts were clipped for 30 minutes by microaneurysm clips, then the mice were fed for another 24 hours for the construction of IRI-induced AKI model. To construct AKI-to-CKD model, bilateral renal ischemia of 35 minutes, followed by reperfusion for 2 weeks was used. To construct cisplatin-induced AKI model, 30 mg/kg of cisplatin was intraperitoneally injected into mice for 3 days as described in previous studies (Li et al., 2018).

To estimate the therapeutic effect of OA, mice were injected with control or OA (20 mg/kg) by tail vein after IRI surgery (Hu et al., 2017) or intraperitoneal cisplatin injection (Li et al., 2018). As reported, the kidney tissues and blood samples were collected (Hu et al., 2017, Li et al., 2018). Serum creatinine (Scr) and blood urea nitrogen (BUN) were determined by using assay kits (Nanjing Jiancheng Bioengineering Institute, China).

### Cell Culture

Human proximal tubular epithelial cells (HK-2) were bought from the American Tissue Culture Collection (ATCC, Manassas, VA, USA) and cultured in F12 medium containing 10% fetal bovine serum (Mediatech, Manassas, VA, USA).

### RNA Extraction and quantitative PCR (qPCR)

Total RNA was extracted from the mouse kidneys and HK-2 cells according to the manufacturers’ instruction with an RNA extraction kit (Beyotime Biotechnology, Shanghai, China), which was reversely transcribed into cDNA with RT Master Mix for qPCR kit (MedChemExpress, Monmouth Junction, NJ, USA). qPCR was carried out by using a SYBR Green qPCR kit (MedChemExpress). The specific primers were listed in Supplementary Table 1 and 2.

### Mitochondrial DNA (mtDNA) Copy Number Detection

Using the DNA of HK-2 cells as a template, the mtDNA copy number was detected by qPCR. The target gene mitochondria-encoded cytochrome c oxidase 2 (MT-CO2) and the internal reference glycerol-3-phosphate dehydrogenase (GAPDH) were detected respectively. The specific primers were listed in Supplementary Table 2.

### Western Blot

Protein extraction and western blot analysis were performed as previously described (Huang et al., 2020, Huang et al., 2020). The signals were detected by an enhanced

chemiluminescence (ProteinSimple, Los Angeles, CA, USA). The primary antibodies were listed as follows: LC3 (12741; CST), P62 (5114; CST), Fibronectin (26836; CST), and α-SMA (48938; CST), Bcl-2 (3498; CST), HIF1α (36169; CST), SATT3 (9139; CST), Bax (199677; Abcam), PPARα (227074; Abcam), BNIP3 (109362; Abcam), COX IV (14744; Abcam).

**Cell Viability Assay**

After OA treatment of HK-2 cells for 48 hours, Cell Counting Kit-8 (CCK-8) solution (Beyotime Biotechnology, C0037) was added, co-incubating at 37 °C for another 1 hour, and the cell viability was analyzed by using Microplate reader (Thermo Fisher Scientific, Waltham, MA, USA).

**Apoptosis Analysis**

After the treatment for indicated times, HK-2 cell solutions were harvested for staining with Annexin V-FITC/PI for 15 minutes using an Apoptosis Assay Kit (Beijing Labgic Technology, China), and apoptosis was measured by flow cytometry (Gallios, BD Biosciences).

**Reactive oxygen species (ROS) Detection**

Adherently cultured cells were incubated with 10 μM DCFH-DA (Beyotime Biotechnology, S0033S) at 37°C for 20 minutes. Cells were collected for numerical analysis by flow cytometer (Gallios, BD Biosciences).

**JC-1 Determination**

HK-2 cells were treated with the enhanced mitochondrial membrane potential assay Kit (JC-1) (Beyotime Biotechnology, C2003S) at 37°C for 20 minutes and collected for flow cytometry analysis (Gallios, BD Biosciences).

**ATP Measurement**

The level of ATP was detected according to the manufacturer's instructions with an enhanced ATP assay kit (Beyotime Biotechnology, S0027).

**Transfection of BNIP3 siRNA**

To verify whether OA maintained mitochondrial homeostasis by inducing BNIP3 expression, the constructed BNIP3 siRNAs (Biomics Biotechnology, Chongqing, China) were transfected into HK-2 cells by Lipofectamine 3000 (Invitrogen, Carlsbad, CA, USA). The BNIP3 siRNA and Negative control siRNA were listed in Supplementary Table 3.

**HBAD-mRFP-EGFP-LC3 assay**

HK-2 cells were infected with HBAD-mRFP-EGFP-LC3 adenovirus (Hanbio

Biotechnology, Shanghai, China), according to the manufacturer’s protocol. Then, the cells under normoxia or HR conditions were treated with OA for 24 hours, and examined with a confocal microscope (Zesis).

**Chromatin immunoprecipitation (ChIP) assay**

The procedure for the CHIP experiment was described in our previous study (Huang et al., 2020). Briefly, the sonicated DNA was immunoprecipitated with 2 μg PPARα antibody, and then amplified by PCR and qPCR, whose primers were listed in Supplementary Table 4.

**Statistical Analysis**

All data were presented as mean ± SEM. Unpaired *t* test or one-way analysis of variance (ANOVA) with Tukey’s test were used for statistical analysis. Statistically significant was defined as $P < 0.05$.

## REFERENCES

Hu, M. C., Shi, M., Gillings, N., Flores, B., Takahashi, M., Kuro, O. M., et al. (2017). Recombinant alpha-Klotho may be prophylactic and therapeutic for acute to chronic kidney disease progression and uremic cardiomyopathy. *Kidney Int*. 5, 1104-1114. dio: 10.1016/j.kint.2016.10.034

Huang, Y., Wang, S., Zhou, J., Liu, Y., Du, C., Yang, K., et al. (2020). IRF1-mediated downregulation of PGC1alpha contributes to cardiorenal syndrome type 4. *Nat Commun*. 1, 4664. dio: 10.1038/s41467-020-18519-0

Huang, Y., Zhou, J., Wang, S., Xiong, J., Chen, Y., Liu, Y., et al. (2020). Indoxyl sulfate induces intestinal barrier injury through IRF1-DRP1 axis-mediated mitophagy impairment. *Theranostics*. 16, 7384-4400. dio: 10.7150/thno.45455

Li, F., Liu, Z., Tang, C., Cai, J., and Dong, Z. (2018). FGF21 is induced in cisplatin nephrotoxicity to protect against kidney tubular cell injury. *Faseb j*. 6, 3423-3433. dio: 10.1096/fj.201701316R

## 2. Supplementary Figures

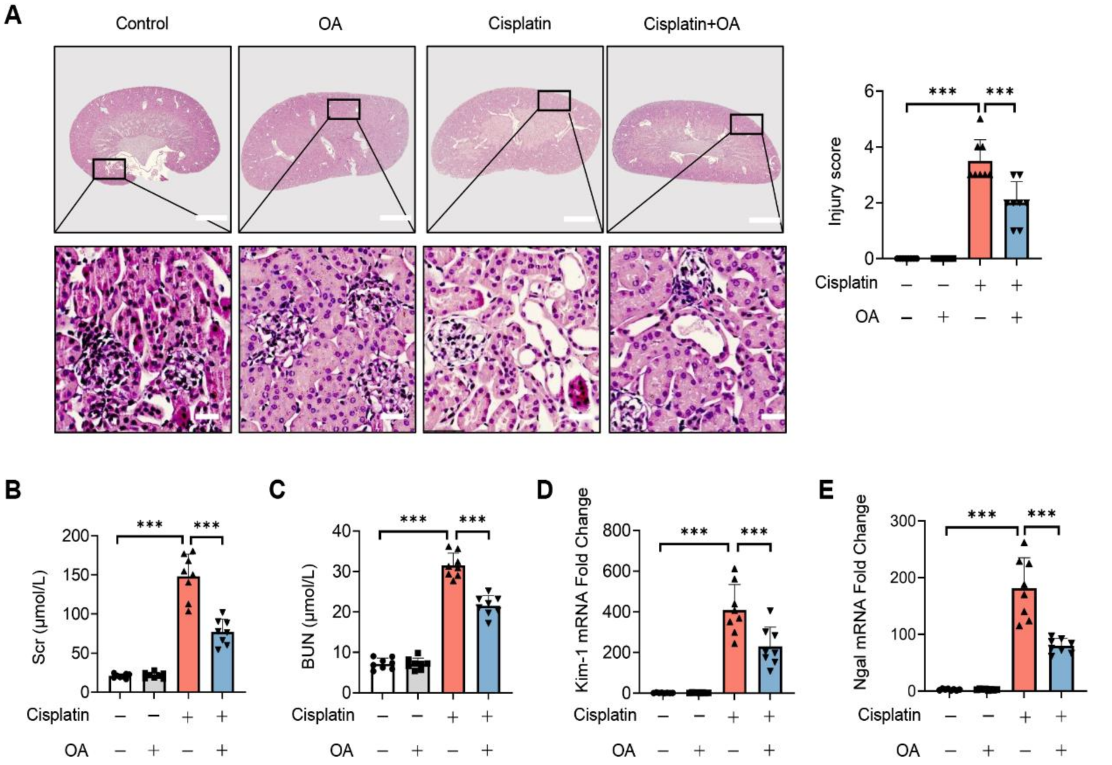


**Supplementary Figure 1.** OA alleviates cisplatin-induced AKI *in vivo*. **(A)** Representative micrographs of HE staining of kidney sections from control and cisplatin-induced AKI mice treated with control or OA for 3 days. Scale bars, 1.25 mm (top) and 50 μm (bottom). **(B, C)** Effects of OA on serum Scr **(B)** and BUN **(C)** (n = 8). **(D, E)** The mRNA levels of Kim-1 **(D)** and Ngal **(E)** were analyzed by qPCR (n = 8). Data are presented as means ± SEM. ****P* < 0.001.

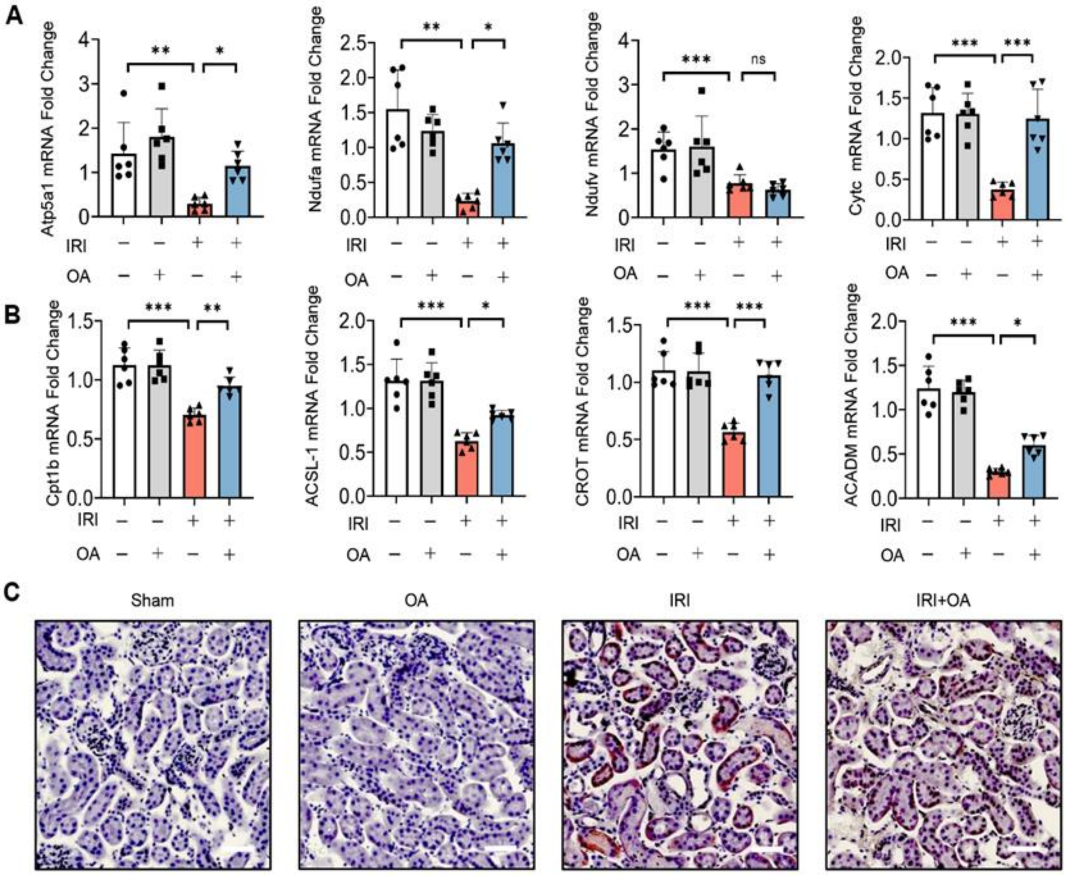


**Supplementary Figure 2.** OA attenuates mitochondrial injury via restoring mitochondrial energy metabolism *in vivo*. **(A, B)** qPCR analysis of the expressions of oxidative phosphorylation and fatty acid oxidation-related genes in kidney tissues from sham and AKI mice treated with control or OA (*n* = 8). **(C)** Representative images of oil red O staining in kidney tissues from sham and AKI mice treated with control or OA. Scale bar, 50 μm. Data are presented as means ± SEM. ns: no significance. *$P$ < 0.05, **$P$ < 0.01, ***$P$ < 0.001.

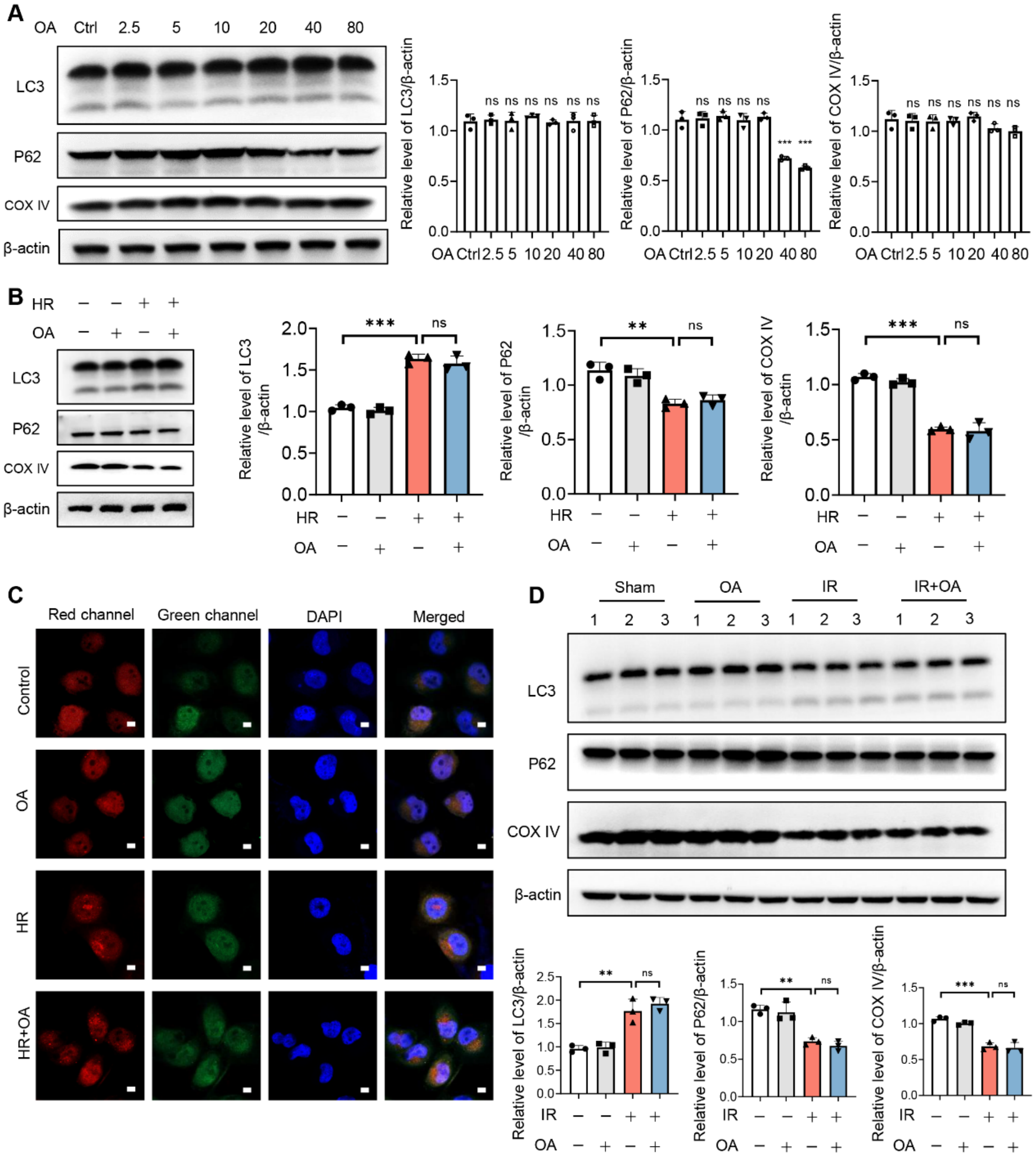


**Supplementary Figure 3.** OA cannot regulate mitophagy in renal tubular epithelial cells. **(A)** Western blot analysis of the expressions of LC3, P62 and COX IV in HK-2 cells treated with control or various doses of OA for 24 hours (n = 3). **(B)** The protein expression of LC3, P62 and COX IV in HK-2 cells exposed to HR followed by incubation with control or OA (10 μM) were analyzed by Western blot (n = 3). **(C)** HK-2 cells were exposed to HR after the infection with HBAD-mRFP-EGFP-LC3 adenovirus and treated with control or OA (10 μM) for 24 hours for confocal microscopy observation. Bar = 10 μm. **(D)** The protein expressions of LC3, P62 and COX IV in the kidney tissues from AKI mice treated with control or OA (20 mg/kg) were analyzed by Western blot (n = 3). Data are presented as means ± SEM. ns: no significance. $^{**}P < 0.01$, $^{***}P < 0.001$.

## 3. Supplementary Tables

**Supplementary Table 1.** The primer sets for mouse

| Gene(mouse) | Primers | Product length |
|---|---|---|
| *β-actin* | Forward: 5′-TGTTACCAACTGGGACGACA-3′<br>Reverse: 5′-GGGGTGTTGAAGGTCTCAAA-3′ | 165 bp |
| *CROT* | Forward: 5′-GGCAAAAAGCTCACCAAGGA-3′<br>Reverse: 5′-CTGAAGAGCGAGCTGAATAAAGG-3′ | 63 bp |
| *Cpt1b* | Forward: 5′-TCTAGGCAATGCCGTTCAC-3′<br>Reverse: 5′-GAGCACATGGGCACCATAC-3′ | 99 bp |
| *ACADM* | Forward: 5′-AGGATGACGGAGCAGCCAATGA-3′<br>Reverse: 5′-GCCGTTGATAACATACTCGTCAC-3′ | 120 bp |
| *ACSL-1* | Forward: 5′-AAATTCTCGCGGTGTCTCCA-3′<br>Reverse: 5′-CACTCGGGGTCTACGTTTCA-3′ | 150 bp |
| *Atp5a1* | Forward: 5'-GCCCTCGGTAATGCTATTGA-3'<br>Reverse: 5'-GCAATCGATGTTTTCCCAGT-3' | 209 bp |
| *Kim-1* | Forward: 5'-ACATATCGTGGAATCACAACGAC-3'<br>Reverse: 5'-ACTGCTCTTCTGATAGGTGACA-3' | 114 bp |
| *Ngal* | Forward: 5'-GCAGGTGGTACGTTGTGGG-3'<br>Reverse: 5'-CTCTTGTAGCTCATAGATGGTGC-3' | 95 bp |
| *Cytc* | Forward: 5′-ACCAAATCTCCACGGTCTGTT-3′<br>Reverse: 5′-GGATTCTCCAAATACTCCATCAG-3′ | 126 bp |
| *Ndufa* | Forward: 5′-ATCCCTTACCCTTTGCCACT-3′<br>Reverse: 5′-CCGTAGCACCTCAATGGACT-3′ | 198 bp |
| *Ndufv* | Forward: 5′-TGTGAGACCGTGCTAATGGA-3′<br>Reverse: 5′-CATCTCCCTTCACAAATCGG-3′ | 217 bp |
| *PPARα* | Forward: 5'-TCAGGGTACCACTACGGAGT-3'<br>Reverse: 5'-CTTGGCATTCTTCCAAAGCG-3' | 206 bp |

**Supplementary Table 2.** The primer sets for human

| Gene(human) | Primers | Product length |
|---|---|---|
| *β-actin* | Forward: 5′-GTGAAGGTGACAGCAGTCGGTT-3′<br>Reverse: 5′-GAAGTGGGGTGGCTTTTAGGA-3′ | 157 bp |
| *Ndufv* | Forward: 5′- ATGAAGGTGACAGCGTGAGG-3′<br>Reverse: 5′- TTCTTGGGTGCTGTCCGC-3′ | 100 bp |
| *Ndufa* | Forward: 5′-GCGCTGTTGCCACTGTT-3<br>Reverse: 5′-AGTCGGGCACGTCCTTC-3 | 103 bp |
| *Atp5a1* | Forward: 5′-ATTTCTCAGTCTACGCCGCA-3'<br>Reverse: 5′-AGACACGCCCAGTTTCTTCA-3' | 147 bp |
| *ACADM* | Forward: 5′- GGCCGTGACCCGTGTATTAT-3'<br>Reverse: 5′-CTGCAGCATCGCCCGAA-3' | 77 bp |
| *Cpt1b* | Forward: 5′- AGGATCTGGGCTTACCTAGAG-3'<br>Reverse: 5′-ACTTGCCCACCATGACTTGA-3' | 144 bp |
| *CROT* | Forward: 5′- GGACACCCTGGTTGTTGCTA-3'<br>Reverse: 5′-AACATCTTTTGCTGCCGCTC-3' | 155 bp |
| *Cycs* | Forward: 5′-CGTTGTGCCAGCGACTAAAAA-3'<br>Reverse: 5′-TTGCCTCCCTTTTCAACGGT-3' | 109 bp |
| *BNIP3* | Forward: 5'-GCTCCCAGACACCACAAGAT-3'<br>Reverse: 5'-TGAGAGTAGCTGTGCGCTTC-3' | 225 bp |
| *ACSL-1* | Forward: 5′-TAAAGACAGATGGGAGGAGACCC-3'<br>Reverse: 5′-ACGTACTGTCGGAAGTCAACC-3' | 170 bp |
| *PPARα* | Forward:5'-GCTATCATTACGGAGTCCACG-3'<br>Reverse: 5'-TCGCACTTGTCATACACCAG-3' | 88 bp |
| *MT-CO2* | Forward: 5´-CAAACCTACGCCAAAATCCA-3´<br>Reverse: 5´-GAAATGAATGAGCCTACAGA-3´ | 164bp |
| *GAPDH* | Forward: 5´-TGACAACAGCCTCAAGAT-3´<br>Reverse: 5´-GAGTCCTTCC ACGATACC-3´ | 195bp |
| *TFAM* | Forward: 5´-CATAAAGCAGGCAGAACTCA-3´<br>Reverse: 5´-GCAACTAAAACCAGCAATCAC-3´ | 107bp |

**Supplementary Table 3.** The sequence sets for siBNIP3 and siNC

| Gene(human) | Sequence |
|---|---|
| *siBNIP3* | Sense: 5′- GGAACACGAGCGUCAUGAA -3′<br>Antisense: 5′- UUCAUGACGCUCGUGUUCC -3′ |
| *siNC* | Sence: 5′- UUCUCCGAACGUGUCACGUTT -3′<br>Antisense: 5′- ACGUGACACGUUCGGAGAATT -3′ |

**Supplementary Table 4.** The primer sets for ChIP

| Gene (human) | Primers | Product length |
|---|---|---|
| *BNIP3* | Forward: 5′- GGCAAATACCTCTTGATGTGCATAAAC -3′<br>Reverse: 5′- ACCTATTCGAGGGAGTCAACTAAAAGT -3′ | 146 bp |
